# Effects of Adipose-derived Stem Cells in the Treatment of Knee Osteoarthritis: A Case Report in Brazil's Unified Health System

**DOI:** 10.1055/s-0041-1733797

**Published:** 2021-10-01

**Authors:** Laynna de Carvalho Schweich-Adami, Roberto Antoniolli da Silva, Adrivanio Baranoski, Candida Aparecida Leite Kassuya, Andréia Conceição Milan Brochado Antoniolli-Silva, Rodrigo Juliano Oliveira

**Affiliations:** 1 Centro de Estudos em Células Tronco, Terapia Celular e Genética Toxicológica – CeTroGen, Hospital Universitário Maria Aparecida Pedrossian – HUMAP/EBSERH, Universidade Federal de Mato Grosso do Sul – UFMS, Campo Grande, MS, Brazil; 2 Programa de Pós-Graduação em Saúde e Desenvolvimento da Região Centro-Oeste, Faculdade de Medicina Dr. Hélio Mandetta – Famed, Universidade Federal de Mato Grosso do Sul – UFMS, Campo Grande MS, Brazil; 3 Ambulatório de Ortopedia e Traumatologia, Hospital Universitário Maria Aparecida Pedrossian – HUMAP/EBSERH, Universidade Federal de Mato Grosso do Sul – UFMS, Campo Grande, MS, Brazil; 4 Faculdade de Ciências da Saúde – FCS, Universidade Federal da Grande Dourados – UFGD, Dourados, MS, Brasil; 5 Programa de Pós-Graduação em Genética e Biologia Molecular, Centro de Ciências Biológicas – CCB, Universidade Estadual de Londrina – UEL, Londrina, PR, Brasil

**Keywords:** cell therapy, inflammation, joint pain, orthopedics, regenerative medicine, Unified Health System

## Abstract

Osteoarthritis (OA) can incapacitate the individual to perform their activities of daily living due to pain. This is an important public health issue that worsens worldwide and in Brazil, since the population goes through an aging process, and has caused increased public spending on the monitoring and maintenance of treatments that can last for years and still not be resolutive. Thus, the search for innovative and effective therapies that can reduce costs becomes necessary. In this context, the present study reports the first application of cell therapy with adipose-derived stem cells in the treatment of cases of OA that are refractory to the conservative treatment, performed in the Brazilian Unified Health System (Sistema Único de Saúde, SUS). The evaluation was performed with the application of the Visual Analog Scale (VAS), the Short Form Health Survey (SF-36) and the Western Ontario and McMaster Universities (WOMAC), specifics for OA evaluation, and also an analysis of the synovial fluid (inflammatory cytokines). The cell therapy improved the scores on the WOMAC, SF-36 and EVA, and reduced the inflammatory process. We observed a decrease of 0.73x in the TNF, of 0,71x in IL-1b, of 0,68x in IL-8, and of 0,70x in IL-10. For IL-6, an increase of 1,48x was observed. Therefore, this cell therapy can be considered promising in aiding the management of this disease, since it improved the patient's pain, decrease inflammatory markers, and enabled the return to activities of daily living, which resulted in an improvement in their quality of life.

## Introduction

Adipose-derived stem cells (ADSCs) have interesting results in the treatment of osteoarthritis (OA). 1 Our preclinical results indicated that ADSCs, by paracrine effect and cell differentiation, can lead to improved repair and regeneration of cartilage. 2 These data were important to start a clinical study which will use ADSCs in the treatment of OA in humans, and to implement innovative therapies at the Cell Processing Center, in partnership with the Orthopedics Service at Universitary Hospital Maria Aparecida Pedrossian (HUMAP/EBSERH), in the city of Campo Grande, state of Mato Grosso do Sul (MS), Brazil.

The present study reports the results of the first cell therapy with ADSCs in a patient of the Brazilian Unified Health System (Sistema Único de Saúde, SUS).

## Case report

A male patient, aged 66 years, weighing 93 kg, and 1,75m, with a diagnosis of medial meniscus rupture in the right knee, with grade III OA (in the Kellgreen and Lawrence classification through radiographic evaluation), and total prosthesis in the left knee. In the clinical evaluation, he reported intermittent pain in the right knee, presented joint cracking with movement, increased knee circumference, and decreased range of motion (ROM), only being able to flex the leg up to 95° without feeling pain. During the interview, we applied the Visual Analog Scale (VAS), the Short Form Health Survey (SF-36), and the Western Ontario and McMaster Universities (WOMAC) osteoarthritis index. The patient was then informed about the treatment with ADSCs and signed the free and informed consent form for the use of cell therapy.

**Fig. 1:**
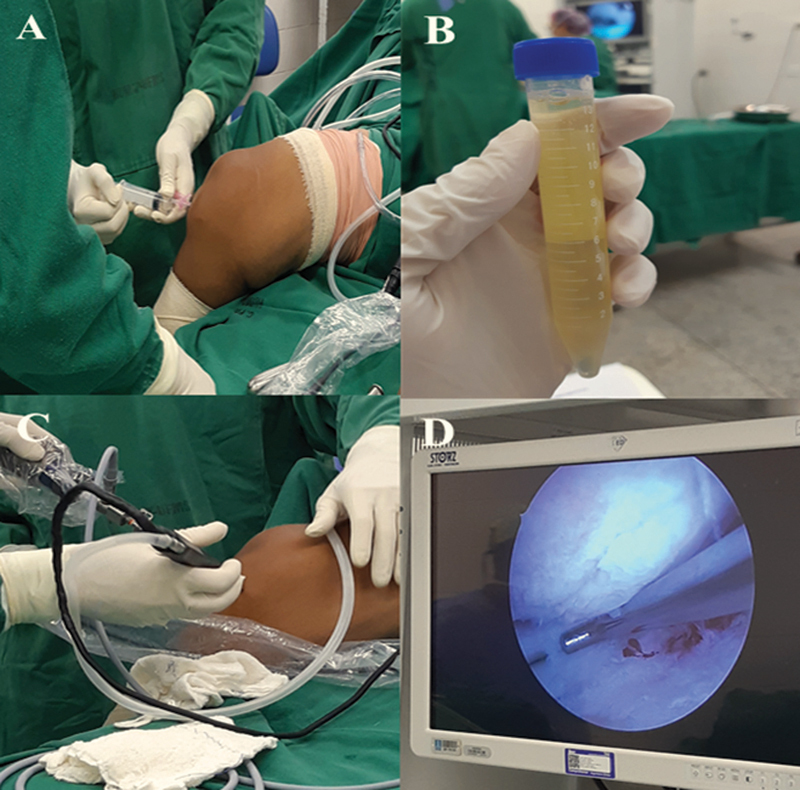
Videoarthroscopy surgical procedure. ( **A** ) Arthrocentesis. ( **B** ) Collected synovial fluid. ( **C** ) Positioning of the surgical instruments and entry sites. ( **D** ) Visualization of articular cartilage to clean debris or areas without leveling.

The following week, a videoarthroscopy surgery was performed according to the routine of the Orthopedics Service (HUMAP/EBSERH) ( Fig. 1C-D ), was performed only cleaning of the joint and light debridement of the affected cartilage. After one week, the patient returned to the hospital for the collection of adipose tissue, a procedure performed by liposuction. 3

**Fig. 2:**
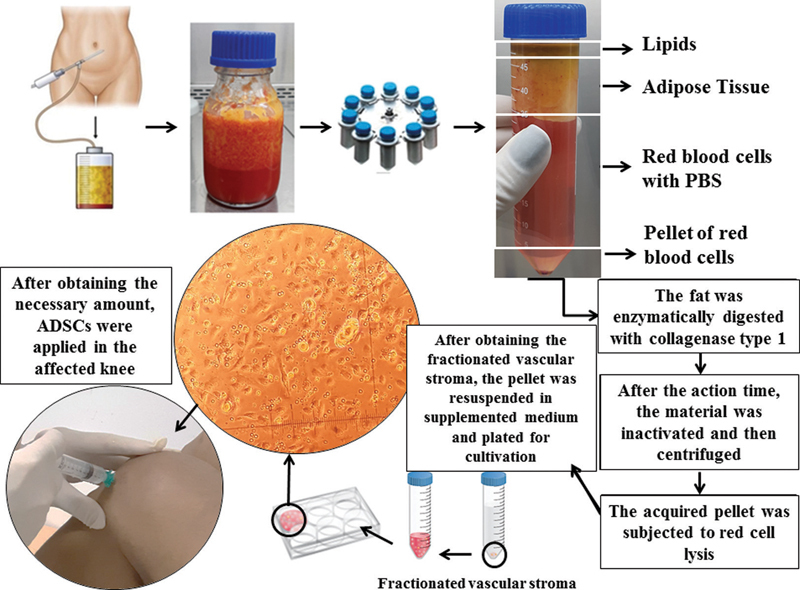
Flowchart of the procedures to obtaining and process lipoaspirate, cultivation of adipose-derived stem cells, and application in the knee with osteoarthritis.

Regarding of the liposuction processing, extraction, culture, characterization (immunophenotyping: CD105, CD90, CD34 and CD133), and cell differentiation (adipogenic, chondrogenic and osteogenic) were performed according to Schweich et al. 4 (2017). The cultivation of ADSCs for transplantation occurred for 25 days (3rd passage), until reaching the necessary amount ( Fig. 2 ). 4 Bacteriological testing was performed before the cell therapy. Then, the patient returned and received an intra-articular injection containing 1 × 10 ^7^ of ADSCs homogenized in 3 mL of saline solution ( Fig. 2 ). A bandage was applied around the treated knee to avoid limb flexion in the first 12 hours.

The collection of synovial fluid occurred in two moments: in the operating room, before the videoarthroscopy ( Fig. 1B ), and after six months of cell therapy. For the analysis of the inflammatory process, we used the CBA Human Inflammatory Cytokines KIT (BD Biosciences, Franklin Lakes, NJ, US), according to the manufacturer's instructions, by flow cytometry (Cytoflex, Beckman Coulter, Inc., Brea, CA, US).

**Fig. 3:**
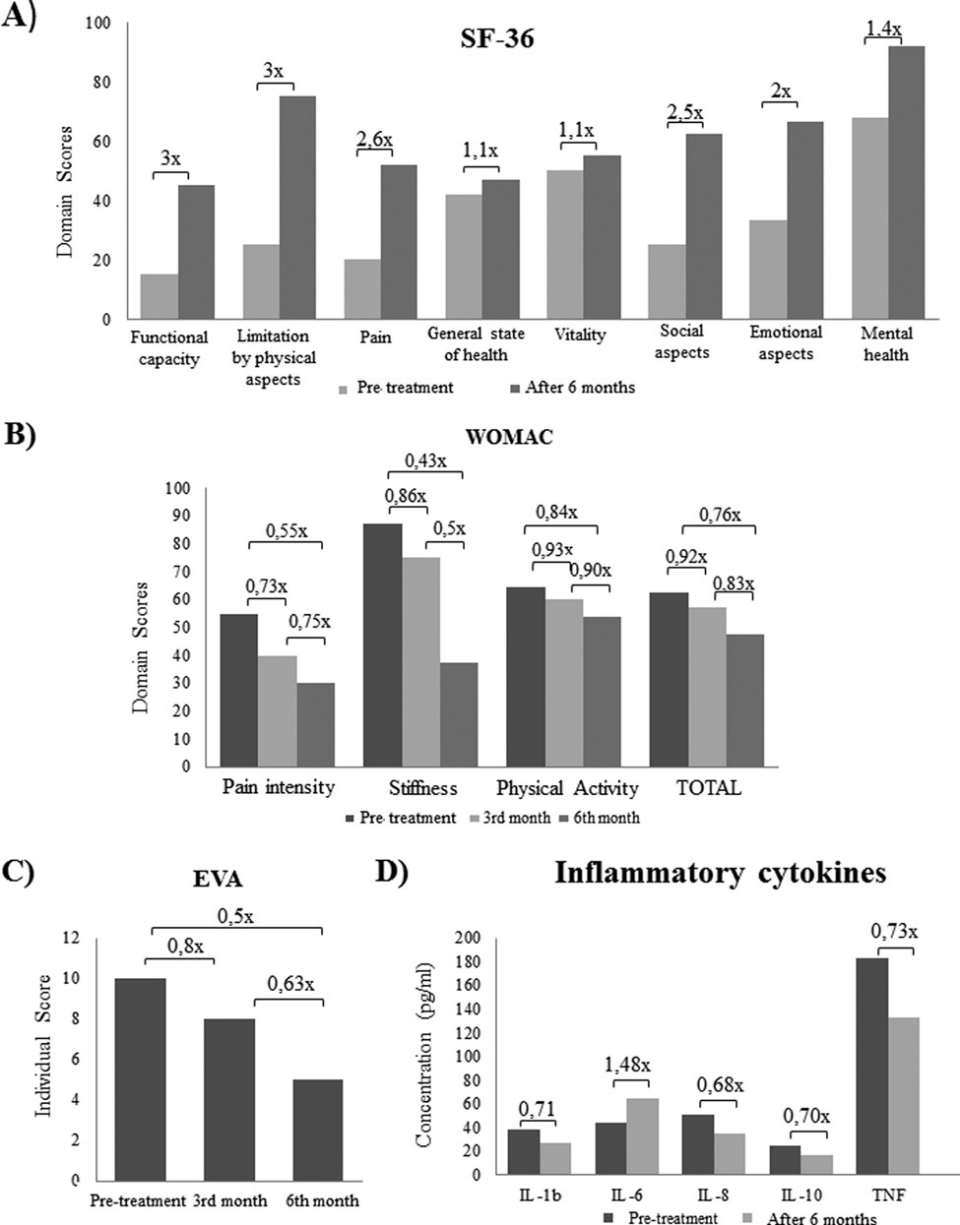
Values acquired after proposed evaluations, in the period before and after treatment with ADSCs. ( **A** ) Score of the SF-36 questionnaire applied before the intervention and after 6 months. ( **B** ) WOMAC questionnaire scores before the intervention, at the 3rd month and at the 6th month. ( **C** ) EVA classification performed before the intervention, in the 3rd month and in the 6th month. ( **D** ) Quantification of inflammatory cytokines before intervention and at the 6th month.

Regarding the results of the cell therapy, we observed that, in the evaluation of the domains of the SF-36 questionnaire, functional capacity and limitation by physical aspects improved by 3x pain by 2,6x, social aspects by 2,5x, the emotional aspects by 2x, mental health by 1,4x and general health and vitality by 1.1x ( Fig. 3A ).

The WOMAC questionnaire indicated a reduction in the score of the domains in both evaluations at three and six months after the cell therapy. In the second evaluation, in relation to the initial condition, the decrease was 0,55x in pain intensity, 0,43x in stiffness, 0,84x for physical activity and 0,76x in the total score ( Fig. 3B ).

The EVA scale showed a descrease of 0,8x and 0,63x for the first (3 months) and second (6 months) assessments, respectively ( Fig. 3C ).

The macroscopic evaluation of the synovial fluid showed improved viscosity, reduction of opacity, and greater homogeneity. The evaluation of inflammatory cytokines showed a decrease of 0,73x of TNF, 0,71x of IL-1b, 0,68x of IL-8 and 0,70x of IL-10. In IL-6, an increase of 1.48x was observed ( Fig. 3D ).

## Discussion

The way of action of ADSCs in the treatment of OA occurs through three different biological effects: cell differentiation, inflammatory modulation (paracrine effect) and mediation of condroprotectors. 5 In the present report, the scores on the SF-36, WOMAC and EVA demonstrate that cell therapy can improve the condition of patients with OA, since it improves the functionality of the affected limb and the patient as a whole, which reflects in the return to activities of daily living and improvements in the overall quality of life, these results corroborate with the current literature. 6 7

In the pathogenesis of OA, the predominance of the IL-6, IL-1b and TNF cytokines stands out. These cytokines have the ability to activate multiple inflammatory pathways, and they may increase disease severity, joint swelling and cartilage destruction. 8 In the present case report, a decrease in the levels of IL-1b, IL-8, IL-10, and mainly TNF, was also observed. With the reduction in these cytokines, it can be suggested that there was a decrease in the local inflammatory process, which aided in the improvement of the degenerative picture of this joint. However, with the specific decrease of IL-10, which is an anti-inflammatory cytokine, 9 it is observed that there is a need for further studies that can describe/understand how ADSCs modulate the inflammatory process in this disease. The only cytokine that increased was IL-6, which is related to the activation of target genes involved in cell differentiation, proliferation and apoptosis. 8 Thus, we infer this increase due to the mild debridement performed during the videoarthroscopy surgery, since it stimulates proliferation/differentiation.

The reduction in the inflammatory process, suggested by the modulation of cytokines, may explain the improvement in the viscosity of the synovial fluid, as well as the reduction in opacity, since the inflammatory process causes an influx of cells into the joint cavity. Therefore, with the reduction in the number of cells, there is a reduction in opacity. In addition to this, and to the increased homogeneity, we observed a reduction in fibrin and remnants of cartilage wear that are also favored by the inflammatory process that has been reduced. 10 These facts are important, since the quality of the synovial liquid is an indicator of the quality of the articular cartilaginous tissue.

We conclude that cell therapy with ADSCs in patients with OA refractory to the conservative treatment can be considered a promising alternative in aiding in the management of this disease, since there is an improvement in pain, and return of the patient to their activities of daily living.
