# Supplementary material for: Effects of Adipose-derived Stem Cells in the Treatment of Knee Osteoarthritis: A Case Report in Brazil's Unified Health System
Source: Rev Bras Ortop (Sao Paulo). 2021 Oct 1;59(3):e471–4. doi: 10.1055/s-0041-1733797 (PMC11193581; doi:10.1055/s-0041-1733797)
Supplement: Related texts

# Research article: Efeitos das células-tronco mesenquimais no tratamento da osteoartrite de joelho: Um relato de caso no Sistema Único de Saúde do Brasil

**Keywords:** dor articular, inflamação, medicina regenerativa, ortopedia, Sistema Único de Saúde, terapia celular

## Abstract

A osteoartrite (OA) pode deixar o indivíduo incapacitado para realizar suas atividades da vida diária devido ao quadro álgico. Essa é uma importante questão de saúde pública que se agrava no mundo inteiro e no Brasil, uma vez que a população passa pelo processo de envelhecimento, e isso causa um aumento nos gastos públicos com o acompanhamento e manutenção dos tratamentos que podem perdurar por anos e mesmo assim não serem resolutivos. Assim, torna-se necessária a busca por terapias inovadoras e eficazes que possam reduzir esses custos. Nesse contexto, o presente estudo relata a primeira aplicação de terapia celular com células-tronco mesenquimais do tecido adiposo no tratamento de OA refratária ao tratamento conservador realizada no Sistema Único de Saúde (SUS). Na avaliação, foram usados os instrumentos Escala Visual Analógica (EVA), os questionários de qualidade de vida Short Form Health Survey (SF-36) e Western Ontario and McMaster Universities (WOMAC), específicos para avaliação da OA, e fez-se uma análise do líquido sinovial (citocinas inflamatórias). A terapia celular melhorou as pontuações no WOMAC, SF-36, e EVA, e reduziu o processo inflamatório. Observou-se redução de 0,73 × do TNF, de 0,71 × da IL-1b, de 0,68 × da IL-8, e de 0,70 × da IL-10. Já para a IL-6, observou-se aumento de 1,48 ×. Portanto, considera-se este tipo de terapia celular promissora no auxílio do manejo desta doença, pois melhorou o quadro álgico do paciente, reduziu os marcadores inflamatórios, e possibilitou o retorno às atividades da vida diária, o que resultou em uma melhora de sua qualidade de vida.

## Introdução

As células-tronco mesenquimais (CTMs) do tecido adiposo apresentam resultados interessantes no tratamento de osteoartrite (OA). 1 Os nossos resultados pré-clínicos indicaram que as CTMs, por efeitos parácrinos e de diferenciação celular, podem levar à melhoria do reparo e da regeneração da cartilagem. 2 Estes dados foram importantes para iniciarmos um estudo clínico que utilizará as CTMs no tratamento da OA em humanos, e implantar e implementar terapias inovadoras no Centro de Processamento Celular, em parceria com o Serviço de Ortopedia, do Hospital Universitário Maria Aparecida Pedrossian (HUMAP/EBSERH), em Campo Grande, Mato Grasso do Sul (MS), Brasil.

O presente estudo relata os resultados da primeira terapia celular com CTMs do tecido adiposo em um paciente do Sistema Único de Saúde (SUS) no Brasil.

## Relato de caso

Paciente do sexo masculino, com 66 anos, 93 Kg e 1,75 m de altura, com diagnóstico de rotura de menisco medial no joelho direito com OA grau III (na classificação de Kellgren e Lawrence pela análise radiográfica), e prótese total no joelho esquerdo. Na avaliação clínica, o paciente relatou dor intermitente no joelho direito, e apresentou crepitação articular durante a movimentação, aumento da circunferência do joelho, e diminuição da amplitude de movimento (ADM), sendo somente capaz de fletir a perna até 95° sem doer. Durante a entrevista, foram aplicados a Escala Visual Analógica (EVA), e os questionários de qualidade de vida Short Form Health Survey (SF-36) e Western Ontario and McMaster Universities (WOMAC), este específico para OA. O paciente foi então esclarecido sobre o tratamento com CTMs, e assinou o termo de consentimento livro e esclarecido para a utilização da terapia celular.

Na semana seguinte, a cirurgia de videoartroscopia foi realizada conforme a rotina do Serviço de Ortopedia do Hospital. Foi realizada a limpeza da articulação e debridamento leve da cartilagem afetada ( Fig. 1C-D ). Após uma semana, o paciente retornou ao hospital para a coleta de tecido adiposo, realizada por lipoaspiração. 3

**Fig. 1:** Procedimento cirúrgico de videoartroscopia. ( **A** ) Artrocentese. ( **B** ) Líquido sinovial coletado. ( **C** ) Posicionamento dos instrumentos cirúrgicos e locais de entrada. ( **D** ) Visualização da cartilagem articular para limpeza de vestígios ou áreas sem nivelamento.

O processamento do lipoaspirado, extração, cultivo, caracterização (imunofenotipagem: [cluster of differentiation, CD, em inglês] CD105, CD90, CD34 e CD133), e diferenciação celular (adipogênica, condrogênica e osteogênica) das CTMs foram realizados segundo Schweich et al. 4 (2017). O cultivo das CTMs para o transplante ocorreu por 25 dias (3a passagem) até atingir a quantidade necessária ( Fig. 2 ). Foi realizado teste bacteriológico antes da terapia celular. Depois, o paciente retornou e recebeu uma injeção intra-articular contendo 1 × 10 ^7^ de CTMs homogeneizadas em 3 ml de solução fisiológica ( Fig. 2 ). Aplicou-se uma bandagem em volta do joelho tratado para evitar a flexão do membro nas primeiras 12 horas.

**Fig. 2:** Fluxograma de procedimentos de obtenção e processamento do lipoaspirado, cultivo das células-tronco mesenquimais, e aplicação no joelho com osteoartrite.

A coleta do líquido sinovial aconteceu em dois momentos: no centro cirúrgico antes da videoartroscopia ( Fig. 1A–B ), e após seis meses da terapia celular. Para a análise do processo inflamatório, utilizou-se o CBA Human Inflammatory Cytokines KIT (BD Biosciences, Franklin Lakes, NJ, EUA), conforme instruções do fabricante, por citometria de fluxo (Cytoflex, Beckman Coulter, Inc., Brea, CA, EUA).

Em relação aos resultados da terapia celular, observamos que, na avaliação dos domínios do questionário SF-36, a capacidade funcional e a limitação por aspectos físicos melhoraram em 3 ×, a dor em 2,6 ×, os aspectos sociais em 2,5 ×, os aspectos emocionais em 2 ×, a saúde mental em 1,4 ×, e o estado geral de saúde e a vitalidade em 1,1 × ( Fig. 3A ).

**Fig. 3:** Valores adquiridos após avaliações propostas no período pré e pós tratamento com CTMs. ( **A** ) Pontuação do questionário SF-36 aplicados antes da intervenção e após 6 meses. ( **B** ) Pontuação do questionário WOMAC antes da intervenção, no 3º mês e no 6º mês. ( **C** ) Classificação de EVA realizada antes da intervenção, no 3º mês e no 6º mês. ( **D** ) Quantificação das citocinas inflamatórias antes da intervenção e no 6º mês.

O questionário WOMAC indicou redução da pontuação dos domínios tanto para a avaliação de três meses quanto para a de seis meses após a terapia celular. Na segunda avaliação, em relação ao quadro inicial, a redução foi de 0,55 × para intensidade da dor, 0,43 × para rigidez, 0,84 × para a atividade física, e 0,76 × na pontuação total ( Fig. 3B ).

A pontuação na EVA demonstrou redução de 0,8 × e 0,63 × para primeira e segunda avaliações, respectivamente ( Fig. 3C ).

A avaliação macroscópica do líquido sinovial demonstrou melhora da viscosidade, redução da opacidade, e maior homogeneidade. A avaliação de citocinas inflamatórias demonstrou redução de 0,73 × do TNF, 0,71 × da IL-1b, de 0,68 × da IL-8, e de 0,70 × da IL-10. Já para IL-6, observou-se aumento de 1,48 × ( Fig. 3D ).

## Discussão

O modo de ação das CTMs no tratamento da OA acontece por meio de três efeitos biológicos diferentes: diferenciação celular, modulação inflamatória (efeito parácrino), e mediação de condroprotetores. 5 Neste relato, os resultados no SF-36, no WOMAC e na EVA demonstram que a terapia celular pode melhorar o quadro do paciente com OA, pois melhora a funcionalidade do membro afetado e do paciente como um todo, o que reflete em retorno às atividades da vida diária e melhoria na qualidade de vida global, esses resultados corroboram com a literatura atual. 6 7

Na patogênese da OA, destacam-se a predominância das citocinas IL-6, IL-1b e TNF. Essas citocinas têm a capacidade de ativar múltiplas vias inflamatórias, podendo aumentar a gravidade da doença, o inchaço das articulações, e a destruição da cartilagem. 8 Neste relato de caso, observou-se também um decréscimo nos níveis de IL-1b, IL-8, IL-10, e principalmente do TNF. Com a redução destas citocinas, pode-se sugerir que houve uma diminuição do processo inflamatório local, o que auxiliou na melhora do quadro degenerativo desta articulação. Porém com a diminuição, em especifico, da IL-10, que é uma citocina anti-inflamatória, 9 observa-se a necessidade de mais estudos que possam descrever/entender como as CTMs modulam o processo inflamatório nessa doença. A única das citocinas que aumentou foi a IL-6, sendo esta relacionada à ativação de genes-alvo envolvidos na diferenciação celular, proliferação e apoptose. 8 Assim, inferimos esse aumento devido ao debridamento leve realizado durante a cirurgia de videoartroscopia, pois ele estimula a proliferação/diferenciação.

A redução do processo inflamatório, sugerido pela modulação das citocinas, pode explicar a melhora da viscosidade do líquido sinovial, bem como a redução da opacidade, pois o processo inflamatório causa influxo de células para a cavidade articular. Logo, com a redução do número de células, ocorre redução da opacidade. Soma-se a isso, e ao aumento da homogeneidade, a redução de fibrina e os restos de desgaste da cartilagem que também são favorecidos pelo processo inflamatório que foi reduzido. 10 Esses fatos são importantes, visto que a qualidade do líquido sinovial reflete na qualidade do tecido cartilaginoso articular.

Infere-se que a terapia celular com CTMs, em pacientes com OA refratária ao tratamento conservador, pode ser considerada uma alternativa promissora no auxílio do manejo dessa doença, pois há melhora do quadro álgico e retorno do paciente às suas atividades da vida diária.
